# Supplementary material for: H/ACA snR30 snoRNP guides independent 18S rRNA subdomain formation
Source: Nat Commun. 2025 May 21;16:4720. doi: 10.1038/s41467-025-59656-8 (PMC12095548; doi:10.1038/s41467-025-59656-8)
Supplement: Supplementary file 1 — Supplementary Information [file 41467_2025_59656_MOESM1_ESM.pdf]

Supplementary Information

H/ACA snR30 snoRNP guides independent 18S rRNA subdomain formation

Paulina Fischer[1,#], Matthias Thoms[2,#], Benjamin Lau[1,3], Timo Denk[2], Maria Kuvshinova[1], Otto Berninghausen[2], Dirk Flemming[1], Ed Hurt[1,*], Roland Beckmann[2,*]

[1] Biochemistry Center, Heidelberg University, Heidelberg, Germany.

[2] Department of Biochemistry, Gene Center, University of Munich, Munich, Germany.

current address:

[3] Molecular Systems Biology Unit, European Molecular Biology Laboratory (EMBL), Meyerhofstrasse 1, 69117 Heidelberg, Germany

[#] These authors contributed equally

[*] Correspondence: ed.hurt@bzh.uni-heidelberg.de (E.H.) and beckmann@genzentrum.lmu.de (R.B.)

Supplementary Information associated with this article includes:

Supplementary Figure 1 to 10

Supplementary Table 1-3

Supplementary References

Supplementary Data 1 (attached datasets)

Supplementary Movie 1

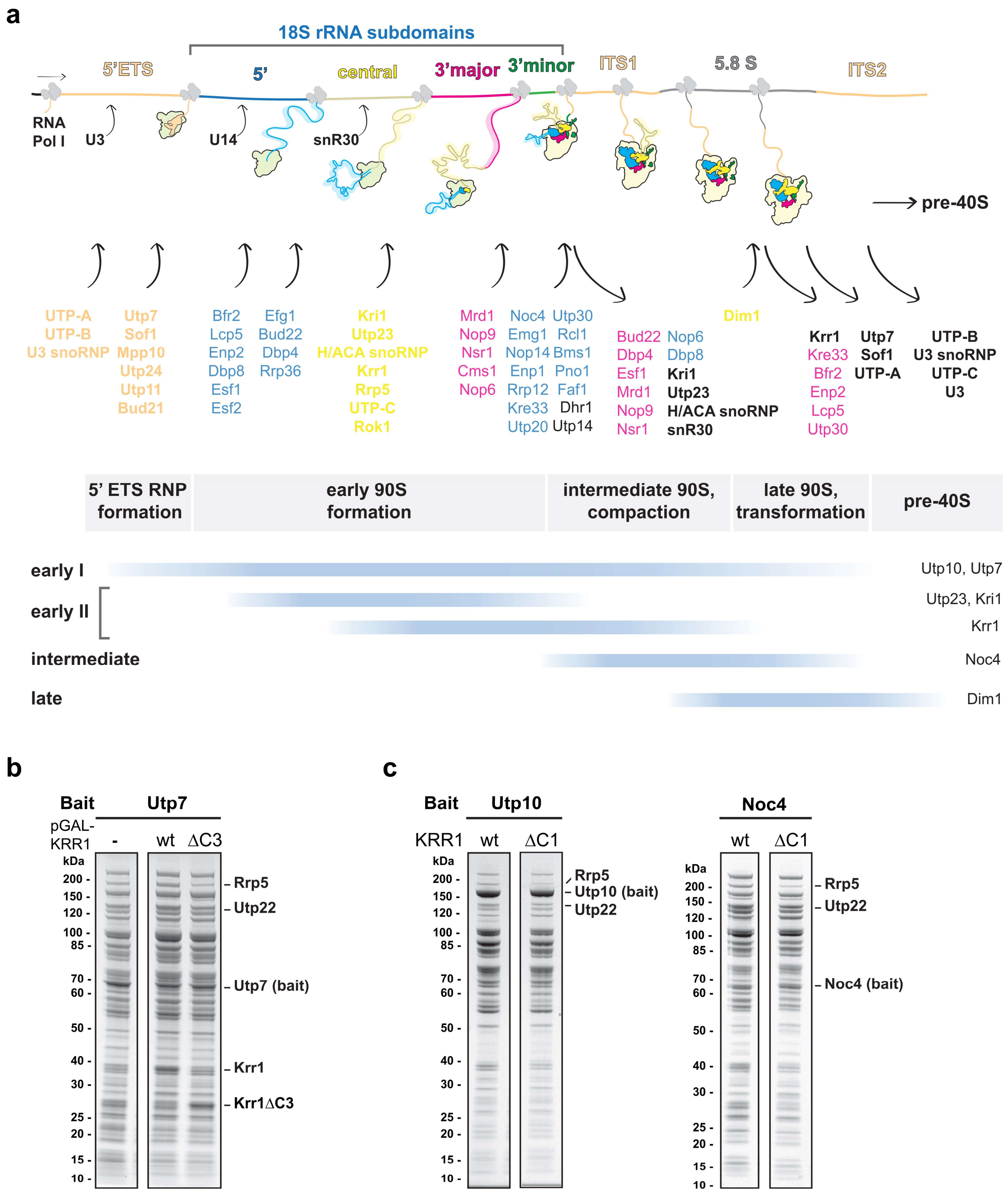

Supplementary Fig. 1. Formation and maturation of 90S pre-ribosomes.

a, Schematic overview of 90S pre-ribosome biogenesis. Assembly factors (AFs) are co-transcriptionally recruited to specific 18S pre-rRNA subdomains of the evolving 90S pre-ribosome. **b**, SDS-PAGE of early 90S particles isolated via Utp7 from cells grown in galactose containing medium either harboring an empty plasmid (-) or galactose inducible wild-type *KRR1* or *krr1*∆C3. **c**, SDS-PAGE of early (Utp10) and intermediate (Noc4) 90S particles isolated from wild-type or *krr1*∆C1 cells. Experiments were performed twice (**b**,**c**).

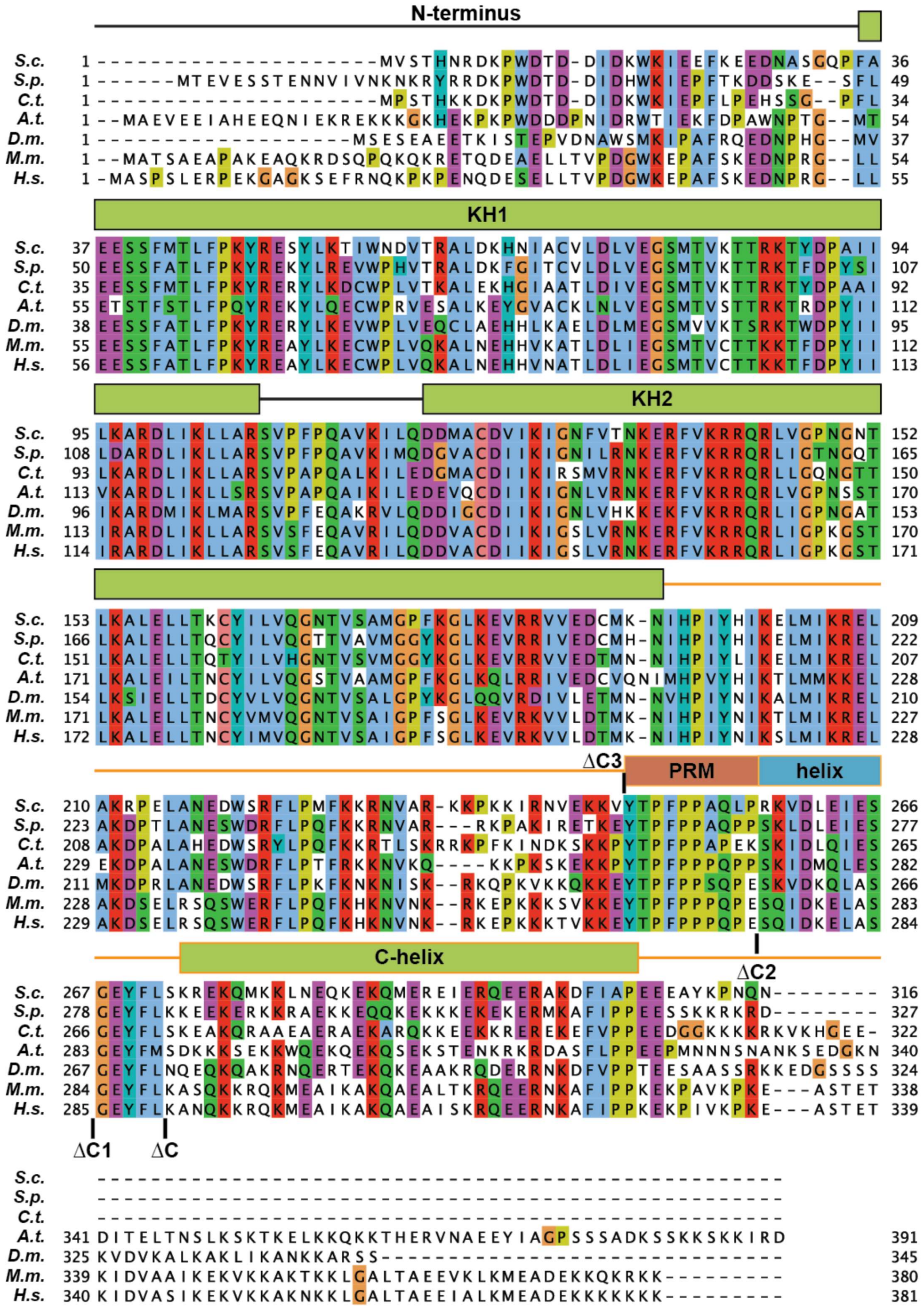

PRM ... proline-rich motif

C-terminus

Supplementary Fig. 2. Krr1 harbors a conserved proline-rich motif within its C-terminal domain.

Multiple sequence alignment of Krr1. C-terminal truncation mutants used in this study as well as the long C-helix and the proline-rich motif (PRM) are indicated. Sequences from *S.c. Saccharomyces cerevisiae, S.p. Schizosaccharomyces pombe, C.t. Chaetomium thermophilum, A.t. Arabidopsis thaliana, D.m. Drosophila melanogaster, M.m. Mus musculus, H.s. Homo sapiens* were aligned with Clustal Omega and displayed with Jalview [1,2].

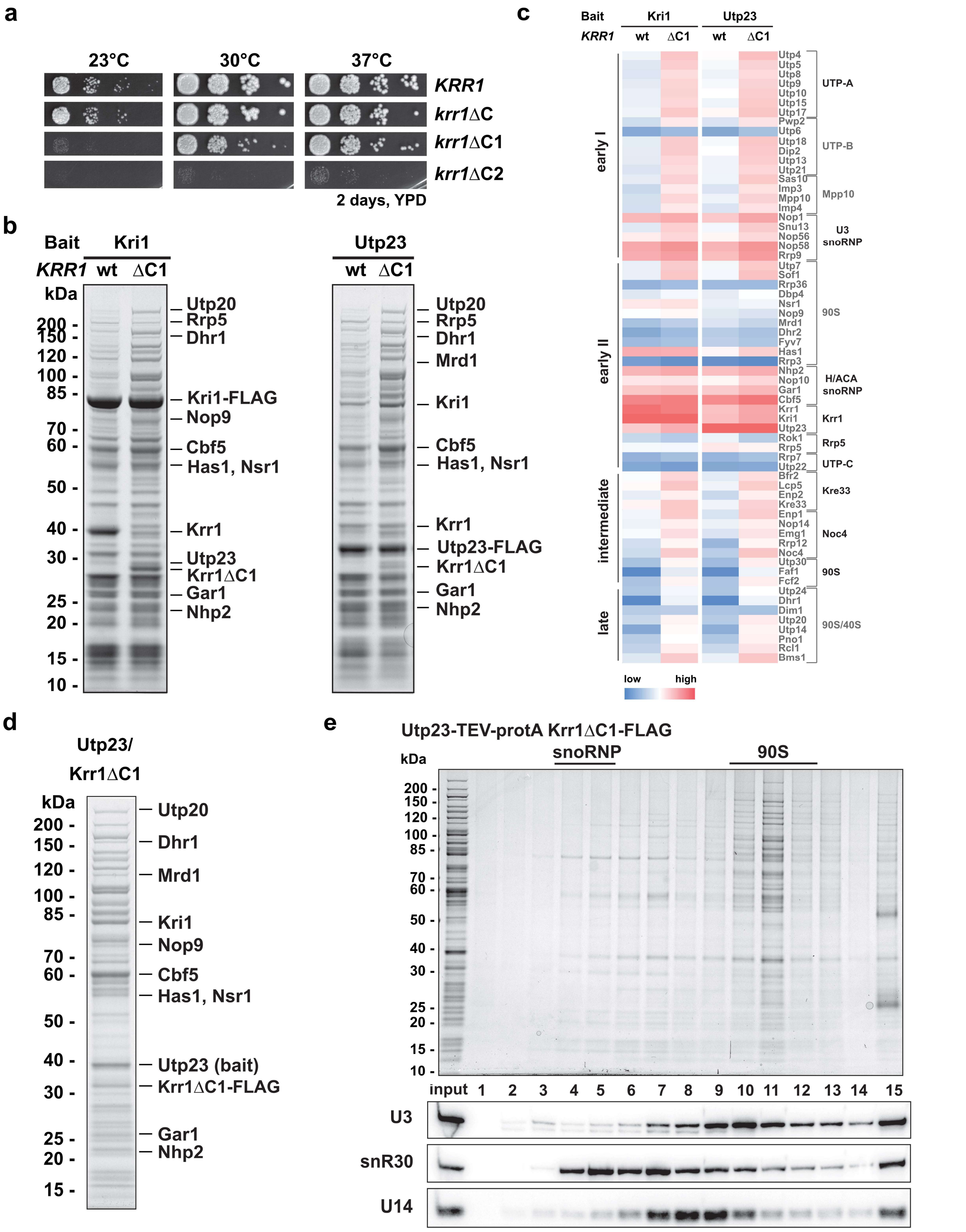

Supplementary Fig. 3. Conserved proline-rich motif of Krr1 affects association of Utp23 and Kri1 with 90S particles.

a, Growth analysis of viable Krr1-CTD truncation mutants at different temperatures. **b**, SDS-PAGE of proteins associated with Kri1 and Utp23 purifications from wt and *krr1*ΔC1 mutant cells. **c**, Heat map of the proteins associated with Kri1 and Utp23 purifications from wt and *krr1*ΔC1 cells as shown in (**b**). Intensity-based iBAQ values were normalized to the bait, and $\log_{10}$ values of the normalized iBAQ values were colored from low (blue) to high (red). Identified proteins are grouped according to their 90S biogenesis module on the right, or according to their dynamic association (**Supplementary Fig. 1a**) on the left. **d**, SDS-PAGE of proteins isolated via Utp23-Krr1ΔC1. **e**, Sucrose gradient analysis of the Utp23-Krr1ΔC1 purification and northern blot analysis of sucrose gradient fractions with probes targeting the snoRNAs snR30, U3 and U14. Experiments were performed more than twice (**a**), twice (**b**, **d**) and once (**c**).

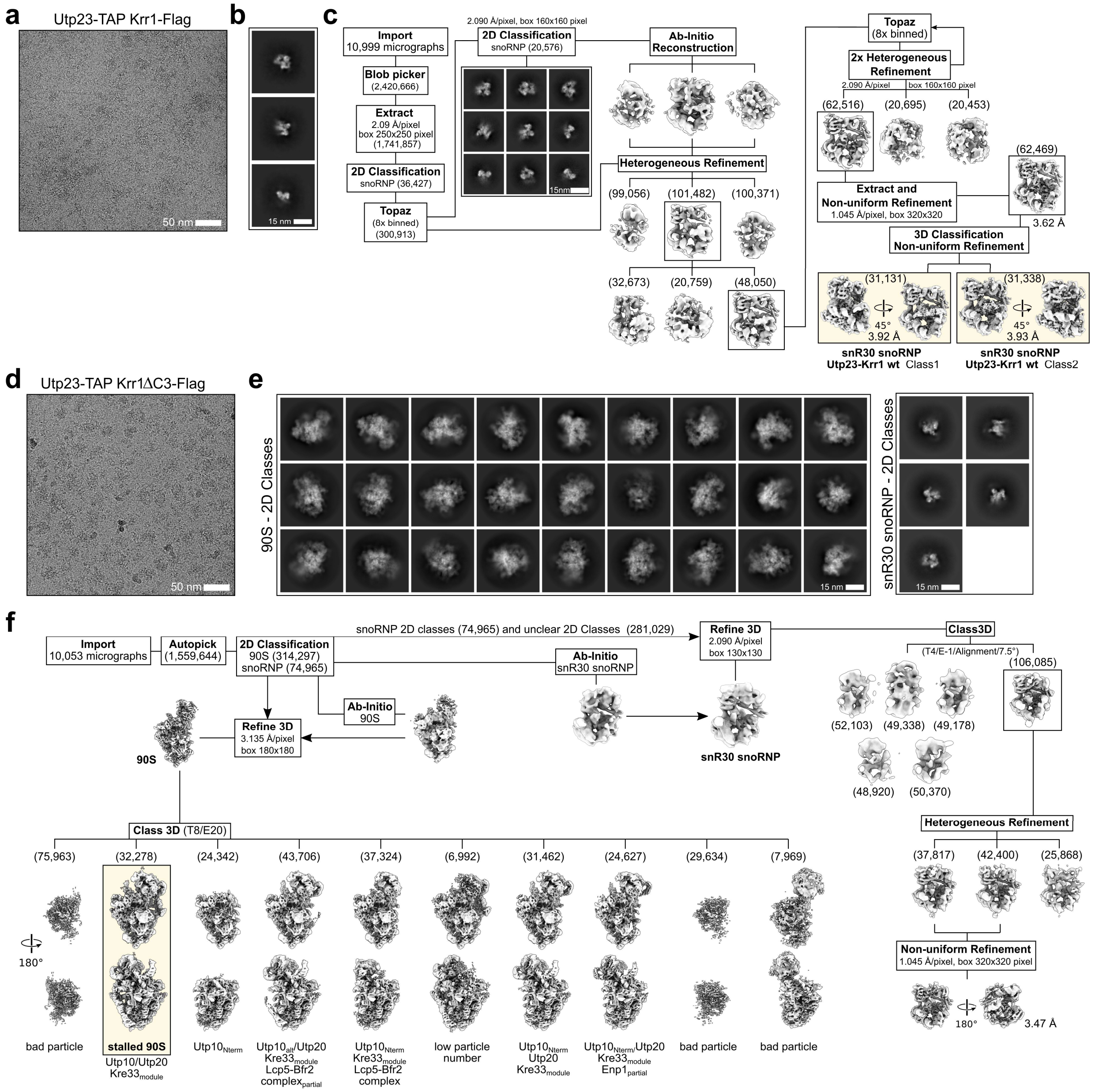

Supplementary Fig. 4. Cryo-EM sorting schemes of the Utp23-Krr1 wt and ΔC3 datasets.

a-**b** and **d**-**e**, Representative electron micrographs (**a**, **d**) and initial 2D class averages (**b**, **e**) of the Utp23-Krr1 wt (**a**, **b**) and the Utp23-Krr1ΔC3 (**d**, **e**) datasets. Scale bars are shown. **c**, **f** Processing schemes of the Utp23-Krr1 wt (**c**) and Utp23-Krr1ΔC3 (**f**) datasets. The Krr1 wt dataset was processed exclusively with cryoSPARC[3], while Relion[4] and cryoSPARC were used for the Utp23-Krr1ΔC3 dataset. **c**, **f** Particle numbers are shown in parentheses, the pixel and box sizes used are given, and the final maps are highlighted. See methods section for details.

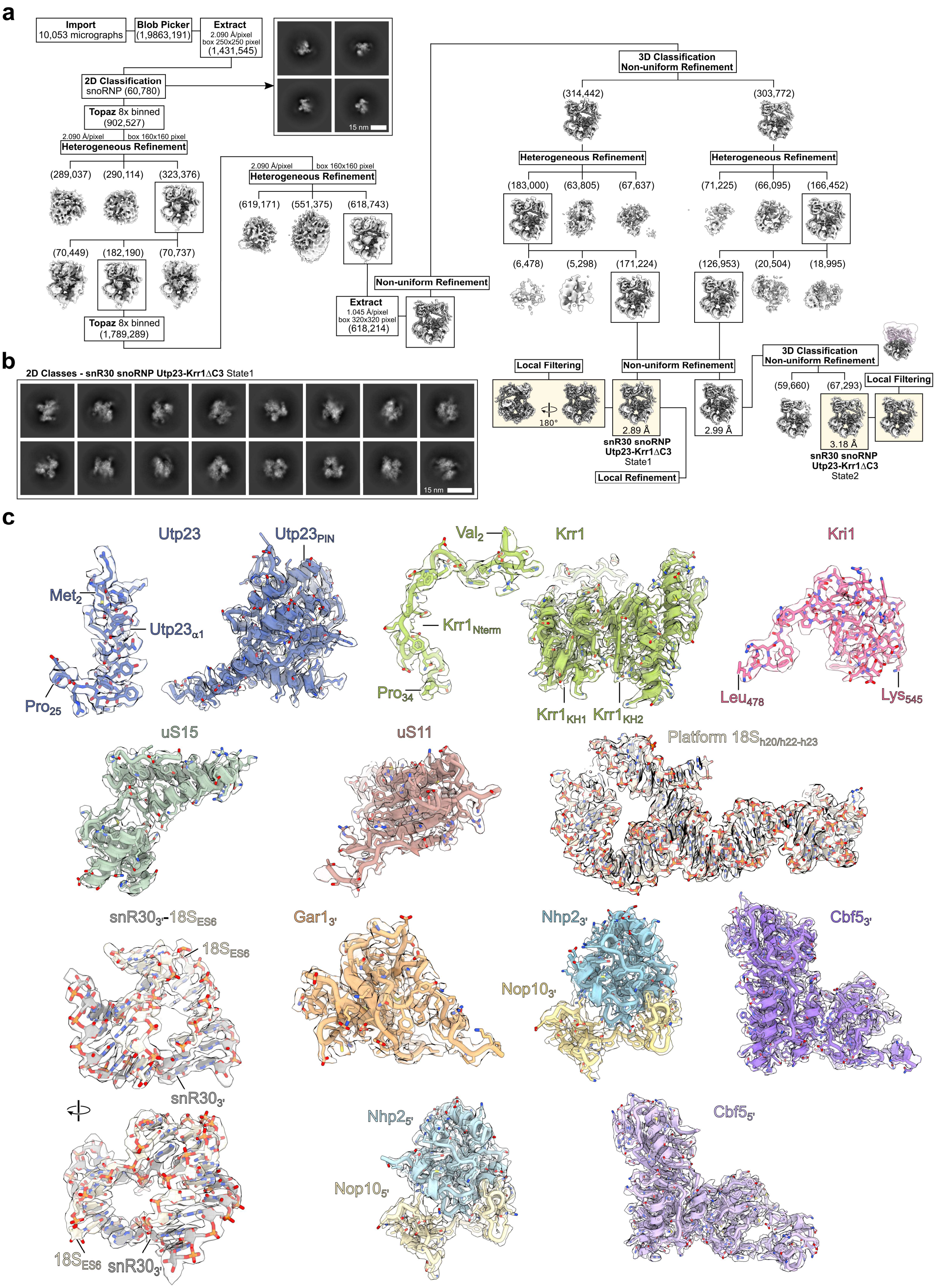
a
Import
10,053 micrographs
Blob Picker
(1,9863,191)
Extract
2.090 Å/pixel
box 250x250 pixel
(1,431,545)
2D Classification
snoRNP (60,780)
15 nm
Topaz 8x binned
(902,527)
2.090 Å/pixel
box 160x160 pixel
Heterogeneous Refinement
(289,037)
(290,114)
(323,376)
(70,449)
(182,190)
(70,737)
Topaz 8x binned
(1,789,289)
2.090 Å/pixel
box 160x160 pixel
Heterogeneous Refinement
(619,171)
(551,375)
(618,743)
Non-uniform Refinement
Extract
1.045 Å/pixel
box 320x320 pixel
(618,214)
3D Classification
Non-uniform Refinement
(314,442)
(303,772)
Heterogeneous Refinement
Heterogeneous Refinement
(183,000)
(63,805)
(67,637)
(71,225)
(66,095)
(166,452)
(6,478)
(5,298)
(171,224)
(126,953)
(20,504)
(18,995)
Local Filtering
Non-uniform Refinement
180°
2.89 Å
2.99 Å
snR30 snoRNP
Utp23-Krr1ΔC3
State1
Local Refinement
3D Classification
Non-uniform Refinement
(59,660)
(67,293)
Local Filtering
3.18 Å
snR30 snoRNP
Utp23-Krr1ΔC3
State2
b
2D Classes - snR30 snoRNP Utp23-Krr1ΔC3 State1
15 nm
c
Utp23
Utp23PIN
Met2
Utp23α1
Pro25
Val2
Krr1
Krr1Nterm
Pro34
Krr1KH1
Krr1KH2
Kri1
Leu478
Lys545
uS15
uS11
Platform 18Sh20/h22-h23
snR303'-18SES6
18SES6
snR303'
Gar13'
Nhp23'
Nop103'
Cbf53'
18SES6
snR303'
Nhp25'
Nop105'
Cbf55'

Supplementary Fig. 5. Cryo-EM sorting scheme for the snR30 snoRNP of the Utp23-Krr1ΔC3 dataset and cryo-EM densities and models of snR30 snoRNP factors.

a, Cryo-EM sorting scheme of the Utp23-Krr1ΔC3 dataset used to obtain the final cryo-EM density maps of snR30 snoRNP Utp23-Krr1ΔC3 State1 and State2. Processing was performed with cryoSPARC. The final maps are highlighted, particle numbers are shown in parentheses and the applied pixel and box sizes are indicated. See methods section for details. **b**, Main 2D class averages for the final snR30 snoRNP Utp23-Krr1ΔC3 State1. Scale bar: 15 nm. **c**, Molecular models and corresponding segmented cryo-EM density maps of the factors identified within the snR30 snoRNP Utp23-Krr1ΔC3 State 1.

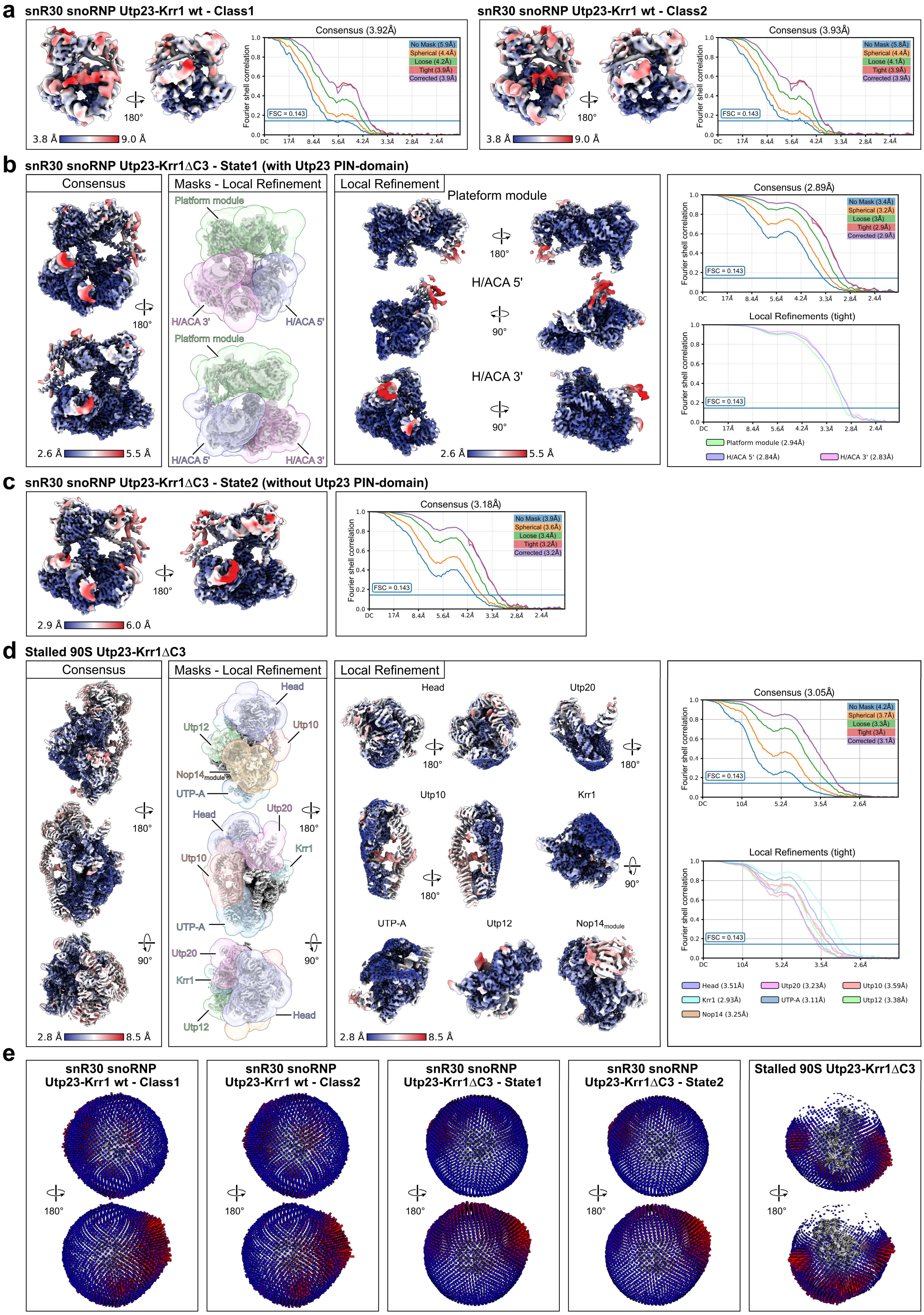
a
snR30 snoRNP Utp23-Krr1 wt - Class1
snR30 snoRNP Utp23-Krr1 wt - Class2
Consensus (3.92Å)
Consensus (3.93Å)
No Mask (5.9Å)
Spherical (4.4Å)
Loose (4.2Å)
Tight (3.9Å)
Corrected (3.9Å)
No Mask (5.8Å)
Spherical (4.4Å)
Loose (4.1Å)
Tight (3.9Å)
Corrected (3.9Å)
FSC = 0.143
Fourier shell correlation
180°
3.8 Å
9.0 Å
b
snR30 snoRNP Utp23-Krr1ΔC3 - State1 (with Utp23 PIN-domain)
Consensus
Masks - Local Refinement
Local Refinement
Platform module
Plateform module
H/ACA 3'
H/ACA 5'
90°
2.6 Å
5.5 Å
Consensus (2.89Å)
No Mask (3.4Å)
Spherical (3.2Å)
Loose (3Å)
Tight (2.9Å)
Corrected (2.9Å)
Local Refinements (tight)
Platform module (2.94Å)
H/ACA 5' (2.84Å)
H/ACA 3' (2.83Å)
c
snR30 snoRNP Utp23-Krr1ΔC3 - State2 (without Utp23 PIN-domain)
Consensus (3.18Å)
No Mask (3.9Å)
Spherical (3.6Å)
Loose (3.4Å)
Tight (3.2Å)
Corrected (3.2Å)
2.9 Å
6.0 Å
d
Stalled 90S Utp23-Krr1ΔC3
Head
Utp12
Utp10
Nop14module
UTP-A
Utp20
Krr1
Consensus (3.05Å)
No Mask (4.2Å)
Spherical (3.7Å)
Loose (3.3Å)
Tight (3Å)
Corrected (3.1Å)
Head (3.51Å)
Utp20 (3.23Å)
Utp10 (3.59Å)
Krr1 (2.93Å)
UTP-A (3.11Å)
Utp12 (3.38Å)
Nop14 (3.25Å)
2.8 Å
8.5 Å
e
snR30 snoRNP Utp23-Krr1 wt - Class1
snR30 snoRNP Utp23-Krr1 wt - Class2
snR30 snoRNP Utp23-Krr1ΔC3 - State1
snR30 snoRNP Utp23-Krr1ΔC3 - State2
Stalled 90S Utp23-Krr1ΔC3

Supplementary Fig. 6. Local resolution, Fourier shell correlation curves and angular distributions.

a, Local resolution filtered cryo-EM density maps and Fourier shell correlation (FSC) curves of the snoRNP classes obtained from the Utp23-Krr1 wt dataset. **b-c**, Local filtered cryo-EM density maps colored according to local resolution and FSC curves of the snoRNP states of the Utp23-Krr1ΔC3 dataset. For State 1 (**b**) masks applied for local refinements and the corresponding local refined maps colored according to the local resolution are shown (middle panels). **d**, Local resolution filtered consensus map of the stalled 90S particle (Utp23-Krr1ΔC3 dataset, left panel). Masks for local refinements and cryo-EM density maps of the local refinements colored according to local resolution (middle). FSC curves of the consensus refinement (top right) and FSC curves of the individual local refinements (bottom right). **e**, Final reconstructions overlaid with 3D representations of the angular distributions, shown in two orientations.

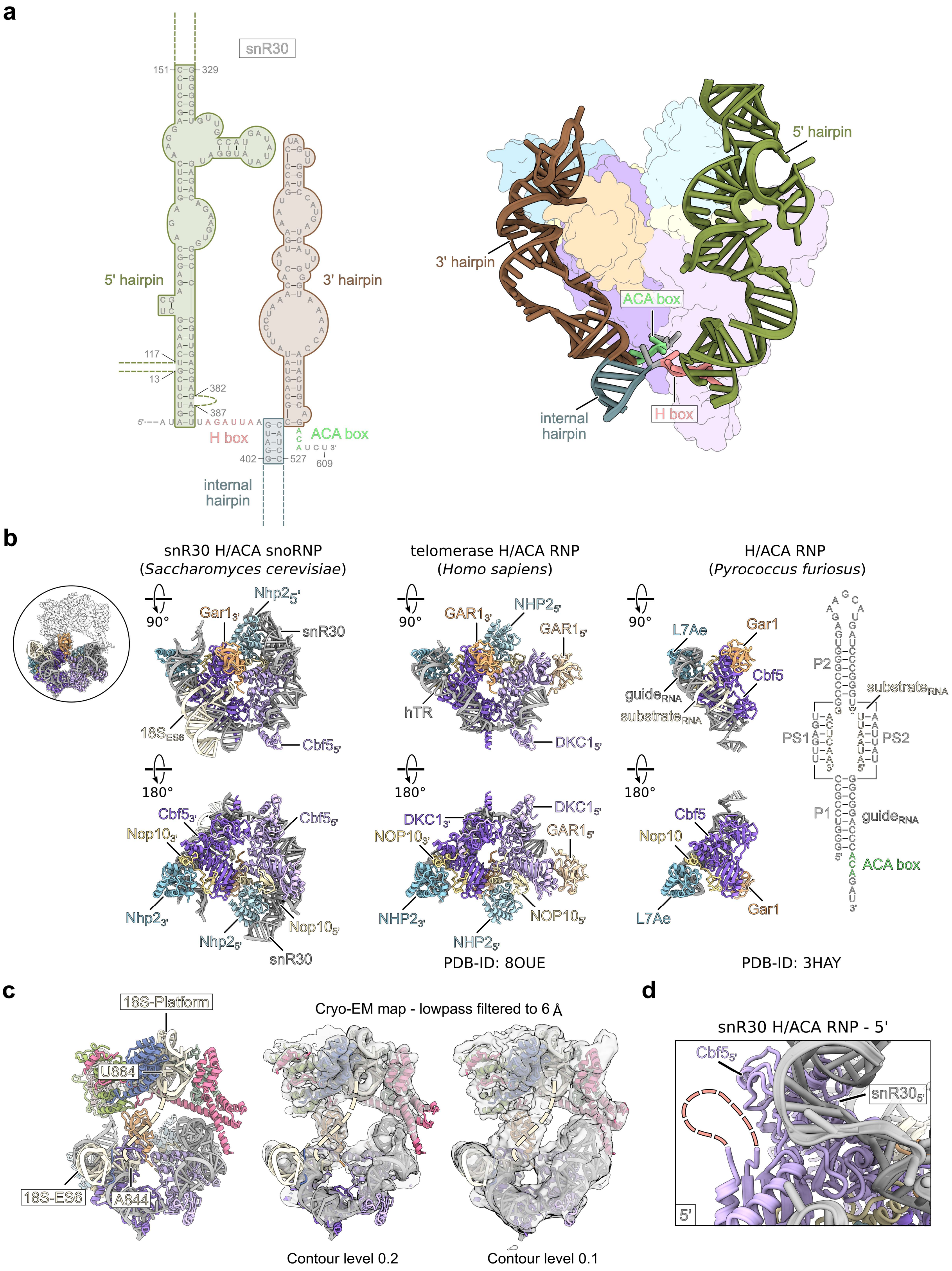

a
snR30
5' hairpin
3' hairpin
H box
ACA box
internal hairpin
151
329
117
13
382
387
402
527
609
b
snR30 H/ACA snoRNP
(Saccharomyces cerevisiae)
telomerase H/ACA RNP
(Homo sapiens)
H/ACA RNP
(Pyrococcus furiosus)
90°
180°
Nhp2 5'
Gar1 3'
snR30
18S ES6
Cbf5 5'
Cbf5 3'
Nop10 3'
Nhp2 3'
Nop10 5'
GAR1 3'
NHP2 5'
GAR1 5'
hTR
DKC1 5'
DKC1 3'
NOP10 3'
NHP2 3'
NOP10 5'
PDB-ID: 8OUE
L7Ae
Gar1
Cbf5
guide RNA
substrate RNA
Nop10
P2
PS1
PS2
P1
ACA box
PDB-ID: 3HAY
c
18S-Platform
U864
18S-ES6
A844
Cryo-EM map - lowpass filtered to 6 Å
Contour level 0.2
Contour level 0.1
d
snR30 H/ACA RNP - 5'
Cbf5 5'
snR30 5'
5'

Supplementary Fig. 7. snR30 structure and comparison of the H/ACA module.

a, Secondary structure of the modeled snR30 regions. The 5', 3' and internal hairpins as well as the H and ACA box consensus sequences are highlighted (left panel). Molecular model of the snR30 snoRNA and surface views of the H/ACA core proteins (right panel). **b**, Comparison of the snR30 H/ACA module with the human telomerase H/ACA module (PDB-ID: 8OUE)[5] and the archaea H/ACA RNP from *Pyrococcus furiosus* (PDB-ID: 3HAY)[6]. The secondary structures of the archaea guide and substrate RNAs are shown. **c**, Model of the snR30 snoRNP and the corresponding lowpass filtered cryo-EM map at the indicated contour levels. The connecting density between the 18S-ES6 bound to the snR30 3' hairpin and 18S-Platform rRNA h22 is visible at lower contour levels and indicated by the dashed line. The boundaries of the 18S molecular model are indicated (left). **d**, Close-up of the putative substrate binding region within the 5' half of the snR30 snoRNP. Here, substrate binding is sterically blocked by the snR30 snoRNA itself, resulting in a delocalized thumb loop of the 5' Cbf5 and the absence of a 5' Gar1 copy. The close-up is shown in the same orientation as the views in Fig. 4g.

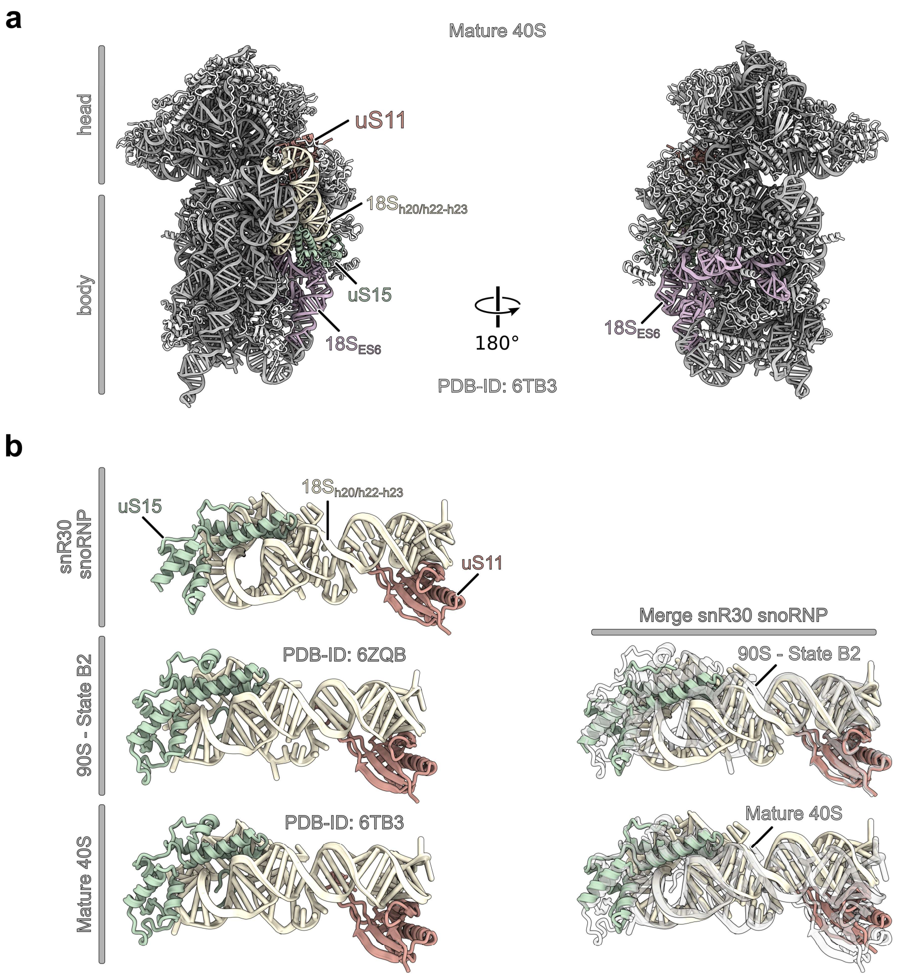

Supplementary Fig. 8. The platform module has a close to mature conformation.

a, Model of the mature 40S subunit (PDB-ID: 6TB3)[7]. Platform helices h20, h22-h23, the complete ES6 and ribosomal proteins uS11 and uS15 are colored and labeled. **b**, Comparison of the platform module within the snR30 snoRNP (Utp23-Krr1ΔC3 State 1), when bound to the 90S particle (State B2, PDB-ID: 6ZQB)[8] and within the mature 40S subunits (PDB-ID: 6TB3) (left panels). Molecular models were rigid body fitted. The snR30 snoRNP platform model is colored and 90S (PDB-ID: 6ZQB) or 40S (PDB-ID: 6TB3) models are shown in transparent gray (right panels).

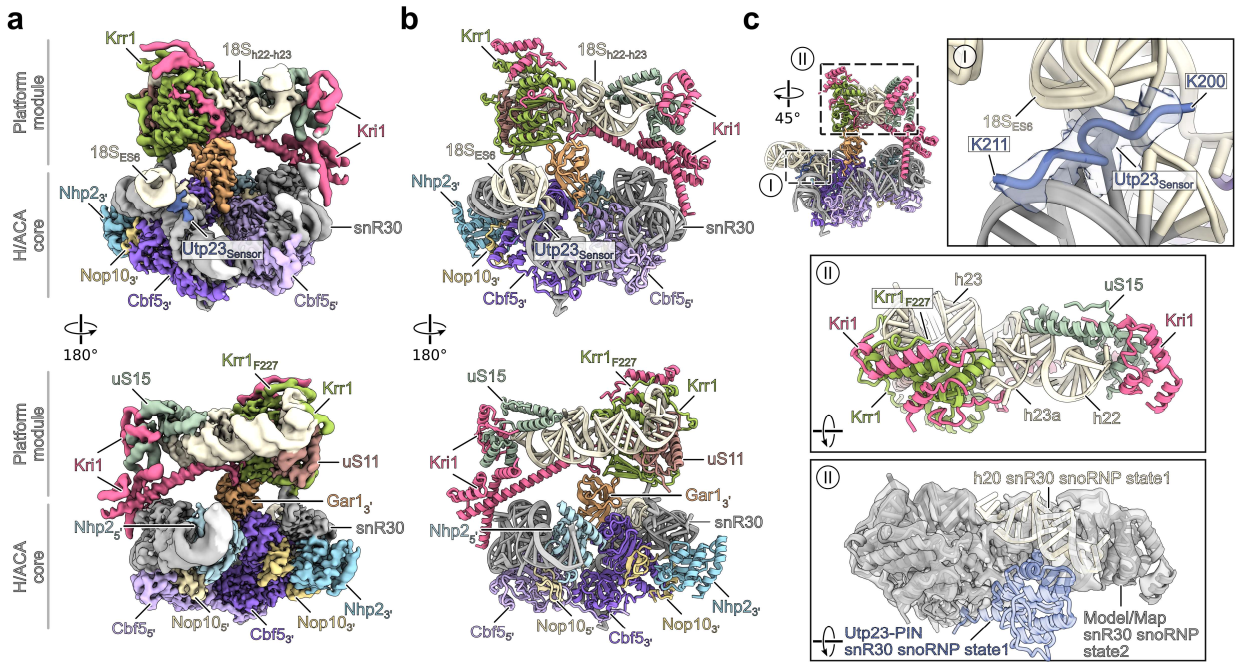

Supplementary Fig. 9. snR30 snoRNP lacking the bound Utp23 PIN domain.

a-**b**, Colored cryo-EM map (**a**) and molecular model (**b**) of the snR30 snoRNP Utp23-Krr1ΔC3 State 2 without a stable bound Utp23-PIN domain. Bound factors, the H/ACA core and the platform module are labeled. **c**, Overview and close-ups of State 2 focusing on the Utp23 interaction regions (upper left). (**I**) Model of the snR30:18S-ES6 three-way junction and cryo-EM density and model of the Utp23-Sensor (aa200-211) are shown. (**II**) Top view showing the molecular model of the snR30 snoRNP Utp23-Krr1ΔC3 State 2 platform module (top panel), which lacks the Utp23 PIN domain, and overlay with the models of 18S rRNA h20 and the Utp23-PIN domain of snR30 snoRNP Utp23-Krr1ΔC3 State 1. Model and density of State 2 are shown in gray and the h20 and the Utp23-PIN domain of State1 are displayed as transparent colored molecular model (bottom panel).

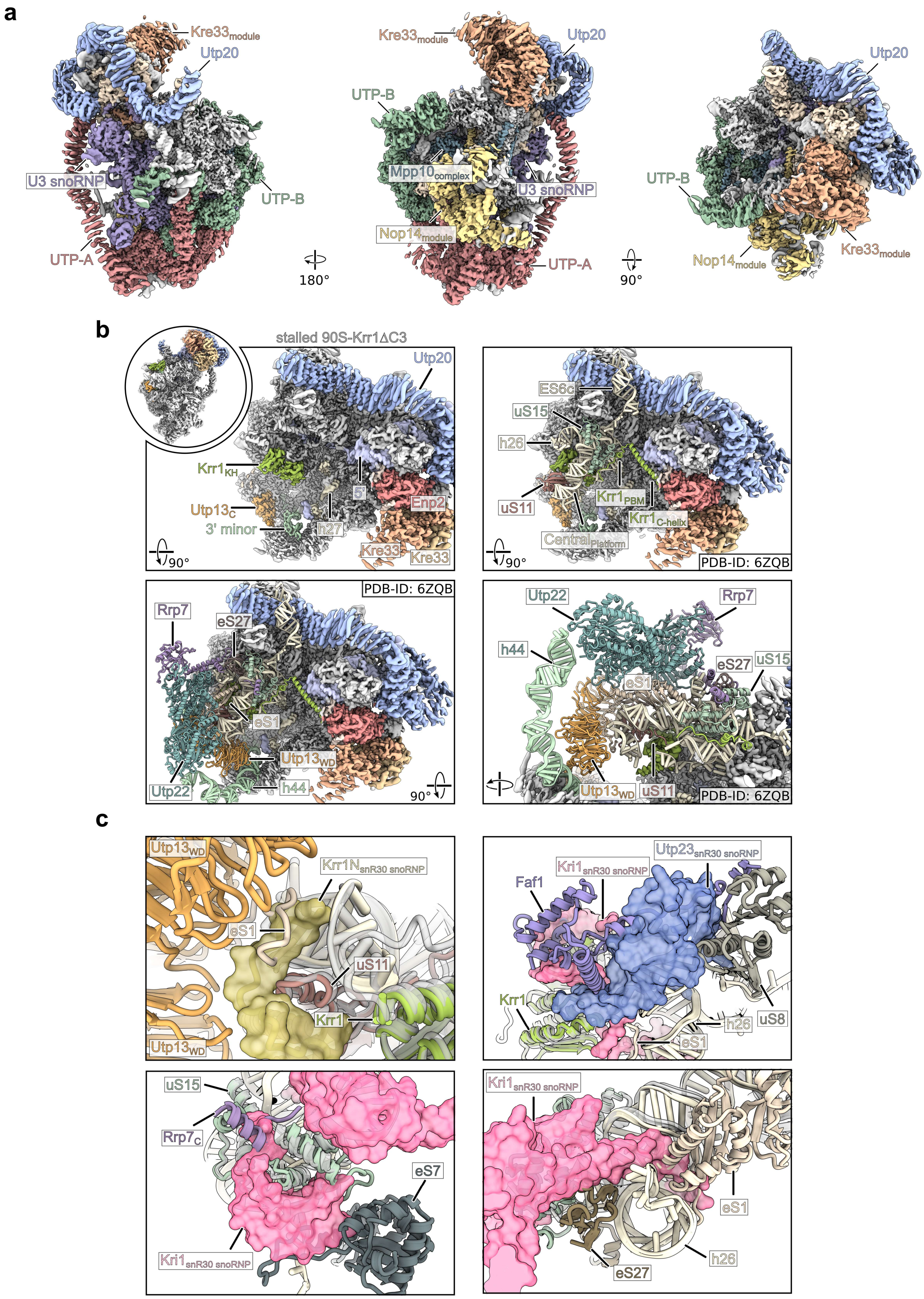
a
Kre33module
Utp20
U3 snoRNP
UTP-B
UTP-A
180°
Kre33module
Utp20
UTP-B
Mpp10complex
U3 snoRNP
Nop14module
UTP-A
90°
Utp20
UTP-B
Nop14module
Kre33module
b
stalled 90S-Krr1ΔC3
Utp20
Krr1KH
5'
Enp2
Utp13C
3' minor
h27
Kre33
Kre33
90°
ES6c
uS15
h26
uS11
Krr1PBM
Krr1C-helix
CentralPlatform
90°
PDB-ID: 6ZQB
PDB-ID: 6ZQB
Rrp7
eS27
eS1
Utp13WD
Utp22
h44
90°
Utp22
Rrp7
h44
eS27
uS15
eS1
Utp13WD
uS11
PDB-ID: 6ZQB
c
Utp13WD
Krr1NsnR30 snoRNP
eS1
uS11
Krr1
Utp13WD
Utp23snR30 snoRNP
Kri1snR30 snoRNP
Faf1
Krr1
h26
uS8
eS1
uS15
Rrp7C
eS7
Kri1snR30 snoRNP
Kri1snR30 snoRNP
eS1
h26
eS27

Supplementary Fig. 10. Structure of the stalled 90S and comparison with the native 90S state B2.

a, Cryo-EM density map of the stalled 90S state purified with Utp23-Krr1ΔC3. Assembly factors and 90S biogenesis modules are colored and labeled. **b**, Overview of the stalled 90S and close-up of the immature central domain region (top left). Depicted factors are labeled for orientation. A Krr1 molecule was identified in its previously observed position within the stalled 90S particle which could be a wild-type copy of Krr1 (note that in the dominant-negative *krr1*ΔC3 mutant, wild-type *KRR1* is still present). Overlay with the molecular model of the state B2 90S (PDB-ID: 6ZQB) showing the central domain rRNA and ribosomal proteins uS11 and uS15 (top right panel) and additionally the interacting UTP-C module (Utp22-Rrp7), the Utp13 WD propellers and rRNA h44 of the 18S 3' minor domain (lower panels). **c**, Magnification views highlighting the steric clashes between the snR30 snoRNP platform module and the 90S. The models of the snR30 snoRNP and the 90S state B2 (PDB-ID: 6ZQB) were rigid body fitted. Molecular models of the snR30 snoRNP are shown as colored transparent surfaces and transparent gray models. The 90S model is depicted as colored molecular model and factors clashing with the snR30 snoRNP structure are labeled.

	**snR30 snoRNP Utp23-Krr1ΔC3** State1 (EMD-50964) (PDB-9G25)	**snR30 snoRNP Utp23-Krr1ΔC3** State2 (EMD-50968) (PDB-9G28)	**snR30 snoRNP Utp23-Krr1 wt** Class1 (EMD-50967)	**snR30 snoRNP Utp23-Krr1 wt** Class2 (EMD-50969)	**Stalled 90S Utp23-Krr1ΔC3** (EMD-50991) (PDB-9G33)
**Data collection & processing**					
Camera	Gatan K2 Summit	Gatan K2 Summit	Gatan K2 Summit	Gatan K2 Summit	Gatan K2 Summit
Magnification	130,000	130,000	130,000	130,000	130,000
Voltage (kV)	300	300	300	300	300
Electron exposure (e^-/Å²)	46.4	46.4	50.0	50.0	46.4
Defocus range (μm)	0.5 - 3.5	0.5 - 3.5	0.5 - 3.5	0.5 - 3.5	0.5 - 3.5
Pixel size (Å)	1.045	1.045	1.045	1.045	1.045
Symmetry imposed	C1	C1	C1	C1	C1
Micrographs collected (no.)	10,053	10,053	10,999	10,999	10,053
Initial particle images (no.)	1,431,545	1,431,545	1,741,857	1,741,857	1,559,644
Final particle images (no.)	171,224	67,293	31,131	31,338	32,278
Map resolution (Å)	2.89	3.18	3.92	3.93	3.05
FSC threshold	0.143	0.143	0.143	0.143	0.143
**Refinement**					
Model resolution (Å)	2.9	3.2			3.1
FSC threshold	0.5	0.5			0.5
Map sharpening B factor ($Å^2$)	-80	-80			-55
Model composition					
Non-hydrogen atoms	24,015	20,876			203,602
Protein residues	2,096	1,951			23,354
Nucleotide residues	367	333			1,581
Ligands	1	0			2
R.m.s deviations					
Bond lengths (Å)	0.003	0.003			0.006
Bond angles (°)	0.726	0.710			0.778
Validation					
Molprobity score	1.15	1.21			1.25
Clash score	3.64	4.33			4.33
Poor rotamers (%)	0.00	0.00			0.02
Ramachandran plot					
Favored (%)	98.88	98.38			97.85
Allowed (%)	1.12	1.62			2.12
Disallowed (%)	0.00	0.00			0.02
Map vs. Model CC (mask)	0.84	0.80			0.84
**Local & Consensus Refinements**	EMD-50958 EMD-50959 EMD-50960 EMD-50961				EMD-50647 EMD-50648 EMD-50649 EMD-50650 EMD-50651 EMD-50652 EMD-50653 EMD-50654

Supplementary Table 1. Cryo-EM data collection, refinement and model statistics.

Plasmid	Reference	Name
P_{KRR1}-*KRR1*-T_{ADH1}, *URA3*, ARS/CEN, *AmpR*	9	pRS316 Krr1wt
P_{KRR1}-*KRR1*-T_{ADH1}, *TRP1*, ARS/CEN, *AmpR*	*9*	pRS314 Krr1wt
P_{KRR1}-*krr1* ΔC-T_{ADH1}, *TRP1*, ARS/CEN, *AmpR*	*9*	pRS314 Krr1ΔC
P_{KRR1}-*krr1* ΔC1-T_{ADH1}, *TRP1*, ARS/CEN, *AmpR*	This study	pRS314 Krr1ΔC1
P_{KRR1}-*krr1* ΔC2-T_{ADH1}, *TRP1*, ARS/CEN, *AmpR*	This study	pRS314 Krr1ΔC2
P_{KRR1}-*krr1* ΔC3-T_{ADH1}, *TRP1*, ARS/CEN, *AmpR*	This study	pRS314 Krr1ΔC3
P_{KRR1}-*krr1* ΔN-T_{ADH1}, *TRP1*, ARS/CEN, *AmpR*	This study	pRS314 Krr1ΔN
P_{UTP7}-*UTP7*-T_{ADH1}, *URA3*, ARS/CEN, *AmpR*	This study	pRS316 Utp7
P_{UTP7}-*UTP7*-T_{ADH1}, *LEU2*, ARS/CEN, *AmpR*	This study	pRS315 Utp7
P_{UTP7}-*UTP7*-FLAG-TEV-ProtA-T_{ADH1}, *LEU2*, ARS/CEN, *AmpR*	This study	pRS315 Utp7-FTpA
$P_{GAL1-10}$-MCS-T_{ADH1}, *LEU2*, 2μ, *AmpR*	This study	pGAL-empty
$P_{GAL1-10}$-*KRR1*-T_{ADH1}, *LEU2*, 2μ, *AmpR*	This study	pGAL-Krr1 Leu
$P_{GAL1-10}$-*KRR1*-T_{ADH1}, *TRP1*, 2μ, *AmpR*	This study	pGAL-Krr1 Trp
$P_{GAL1-10}$-*krr1* ΔC3-T_{ADH1}, *LEU2*, 2μ, *AmpR*	This study	pGAL-Krr1ΔC3 Leu
$P_{GAL1-10}$-*krr1* ΔC3-T_{ADH1}, *TRP1*, 2μ, *AmpR*	This study	pGAL-Krr1ΔC3 Trp
$P_{GAL1-10}$-*krr1*-T_{ADH1}, *TRP1*, 2μ, *AmpR*	This study	pGAL-Krr1ΔN Trp

Supplementary Table 2. Plasmids used in this study

Strain	Reference	Name
ade2-1 ,trp1-1, leu2-3,112, his3-11,15, ura3-1, can1-100	*10*	W303
W303, *krr1*::*HIS3*, [pRS316 *KRR1*]	*10*	Krr1 shuffle
W303, *krr1*::*HIS3*, *utp7*::*hphNT1* [pRS316 *KRR1*, pRS316 *UTP7*]	This study	Krr1-Utp7 double shuffle
W303, *krr1*::*HIS3*, *utp7*::*hphNT1*, *NOC4*-FLAG-TEV-ProtA::*natNT2* [pRS316 *KRR1*, pRS316 *UTP7*]	This study	Krr1-Utp7 double shuffle, Noc4-FTpA
W303, *krr1*::*HIS3*, *UTP10*-FLAG-TEV-ProtA::*kanMX6* [pRS316 *KRR1*]	This study	Krr1 shuffle, Utp10-FTpA
W303, *krr1*::*HIS3*, *UTP7*-FLAG-TEV-ProtA::*natNT2* [pRS316 *KRR1*]	This study	Krr1 shuffle, Utp7-FTpA

Supplementary Table 3. Strains used in this study.

Supplementary References

1 Madeira, F. *et al.* The EMBL-EBI Job Dispatcher sequence analysis tools framework in 2024. *Nucleic Acids Res* **52**, W521-W525 (2024). https://doi.org/10.1093/nar/gkae241

2 Waterhouse, A. M., Procter, J. B., Martin, D. M., Clamp, M. & Barton, G. J. Jalview Version 2--a multiple sequence alignment editor and analysis workbench. *Bioinformatics* **25**, 1189-1191 (2009). https://doi.org/10.1093/bioinformatics/btp033

3 Punjani, A., Rubinstein, J. L., Fleet, D. J. & Brubaker, M. A. cryoSPARC: algorithms for rapid unsupervised cryo-EM structure determination. *Nat Methods* **14**, 290-296 (2017). https://doi.org/10.1038/nmeth.4169

4 Zivanov, J. *et al.* New tools for automated high-resolution cryo-EM structure determination in RELION-3. *Elife* **7** (2018). https://doi.org/10.7554/eLife.42166

5 Ghanim, G. E., Sekne, Z., Balch, S., van Roon, A. M. & Nguyen, T. H. D. 2.7 A cryo-EM structure of human telomerase H/ACA ribonucleoprotein. *Nat Commun* **15**, 746 (2024). https://doi.org/10.1038/s41467-024-45002-x

6 Duan, J., Li, L., Lu, J., Wang, W. & Ye, K. Structural mechanism of substrate RNA recruitment in H/ACA RNA-guided pseudouridine synthase. *Mol Cell* **34**, 427-439 (2009). https://doi.org/10.1016/j.molcel.2009.05.005

7 Buschauer, R. *et al.* The Ccr4-Not complex monitors the translating ribosome for codon optimality. *Science* **368** (2020). https://doi.org/10.1126/science.aay6912

8 Cheng, J. *et al.* 90S pre-ribosome transformation into the primordial 40S subunit. *Science* **369**, 1470-1476 (2020). https://doi.org/10.1126/science.abb4119

9 Cheng, J. *et al.* Thermophile 90S Pre-ribosome Structures Reveal the Reverse Order of Co-transcriptional 18S rRNA Subdomain Integration. *Mol Cell* **75**, 1256-1269 e1257 (2019). https://doi.org/10.1016/j.molcel.2019.06.032

10 Thomas, B. J. & Rothstein, R. Elevated recombination rates in transcriptionally active DNA. *Cell* **56**, 619-630 (1989). https://doi.org/10.1016/0092-8674(89)90584-9
