# Supplementary material for: H/ACA snR30 snoRNP guides independent 18S rRNA subdomain formation
Source: Nat Commun. 2025 May 21;16:4720. doi: 10.1038/s41467-025-59656-8 (PMC12095548; doi:10.1038/s41467-025-59656-8)
Supplement: Supplementary file 2 — Description of Additional Supplementary Files [file 41467_2025_59656_MOESM2_ESM.pdf]

Description of Additional Supplementary Files

File name: Supplementary Data 1
Description: Semi-quantitative Mass Spectrometry results for Figures 1, 2 and Supplementary Figure 3.

File name: Supplementary Movie 1
Description: Structure of the non-canonical snR30 snoRNP showing its bipartite organization.
