# Supplementary material for: H/ACA snR30 snoRNP guides independent 18S rRNA subdomain formation
Source: Nat Commun. 2025 May 21;16:4720. doi: 10.1038/s41467-025-59656-8 (PMC12095548; doi:10.1038/s41467-025-59656-8)
Supplement: Supplementary file 7 — Source Data [file 41467_2025_59656_MOESM7_ESM.pdf]

a

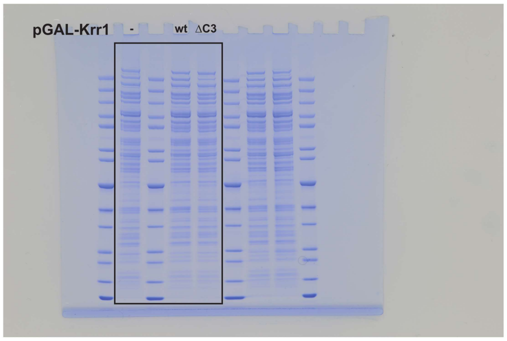

Noc4-FTpA from cells containing empty plasmid (-), expressing pGAL-Krr1wt and pGAL-Krr1ΔC3.
Used in Figure 1e.

b

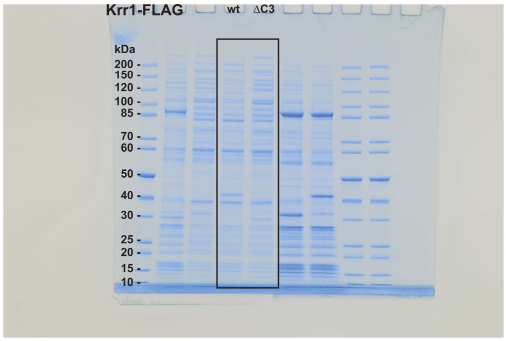

Utp23-TEV-protA and Krr1wt-FLAG or Krr1ΔC3-FLAG.
Used in Figure 2a.

c

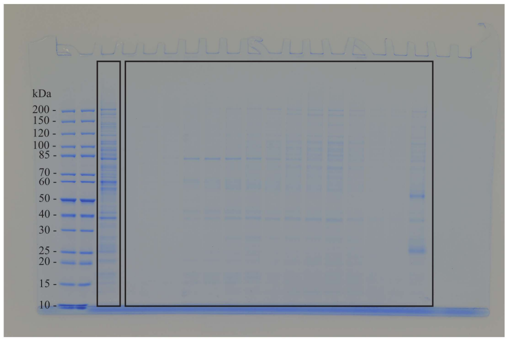

Utp23-TEV-protA and Krr1wt-FLAG on sucrose gradient.
Used in Figure 2c.

d

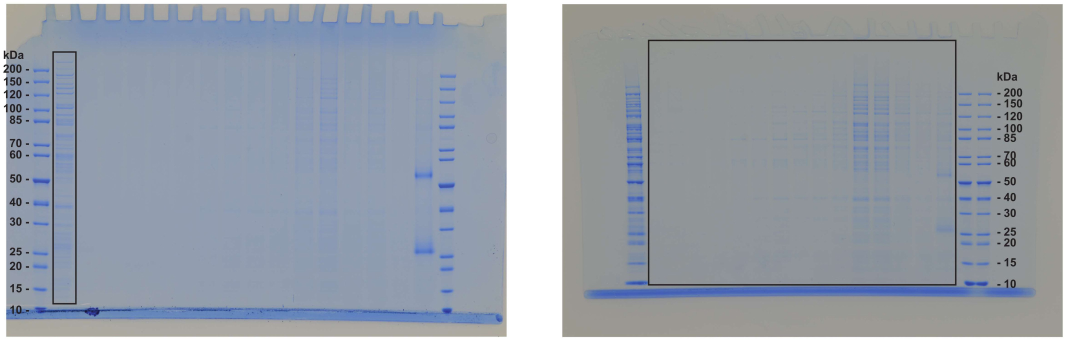

Utp23-TEV-protA and Krr1ΔC3-FLAG on sucrose gradient.
Used in Figure 2e.

Source Data Figure 1 Uncropped images of SDS-PAGE from Main Figures.

Uncropped gel image of final FLAG eluates separated on 4-12% SDS-PAGE after colloidal Coomassie staining. The lines indicate the regions cropped for display in the respective figures. a: Figure 1, b-d: Figure 2.

a

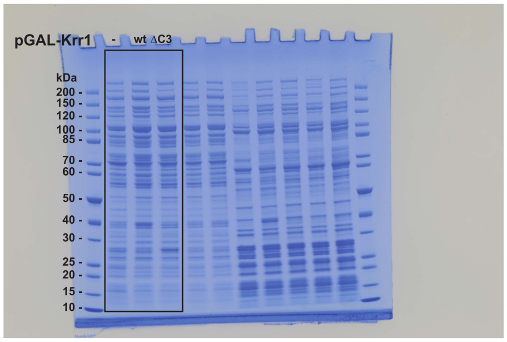

Utp7-FTpA from cells containing empty plasmid, expressing pGAL-Krr1 wt or pGAL-Krr1ΔC3. Used in Supplementary Figure 1b.

b

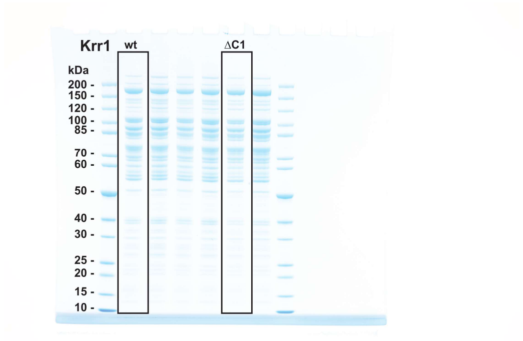

Utp10-FTpA from Krr1wt and Krr1ΔC1 cells. Used in Supplementary Figure 1c.

c

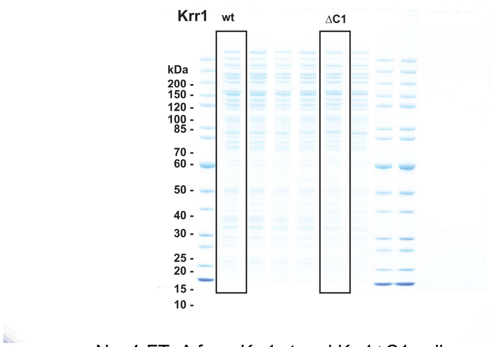

Noc4-FTpA from Krr1wt and Krr1ΔC1 cells. Used in Supplementary Figure 1c.

d

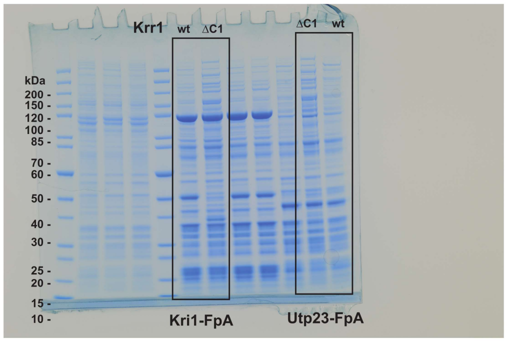

Kri1-FTpA from Krr1wt and Krr1ΔC1 cells, Utp23-FTpA from Krr1wt and Krr1ΔC1 cells. Used in Supplementary Figure 3b.

e

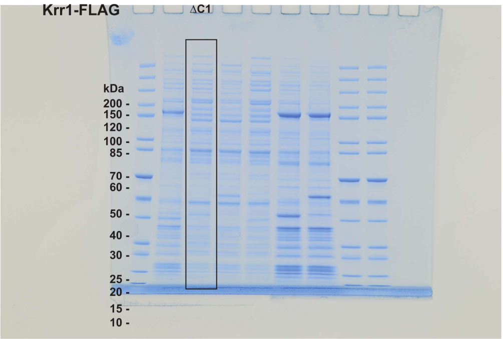

Utp23-TEV-protA and Krr1ΔC1-FLAG. Used in Supplementary Figure 3d.

f

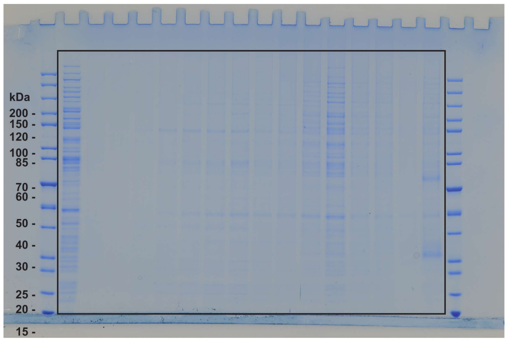

Utp23-TEV-protA and Krr1ΔC1-FLAG. Used in Supplementary Figure 3e.

Source Data Figure 2 Uncropped images of SDS-PAGE from Supplementary Figures.

Uncropped gel image of final FLAG eluates separated on 4-12% SDS-PAGE after colloidal Coomassie staining. a-c: Supplementary Figure 1, d-f: Supplementary Figure 3.

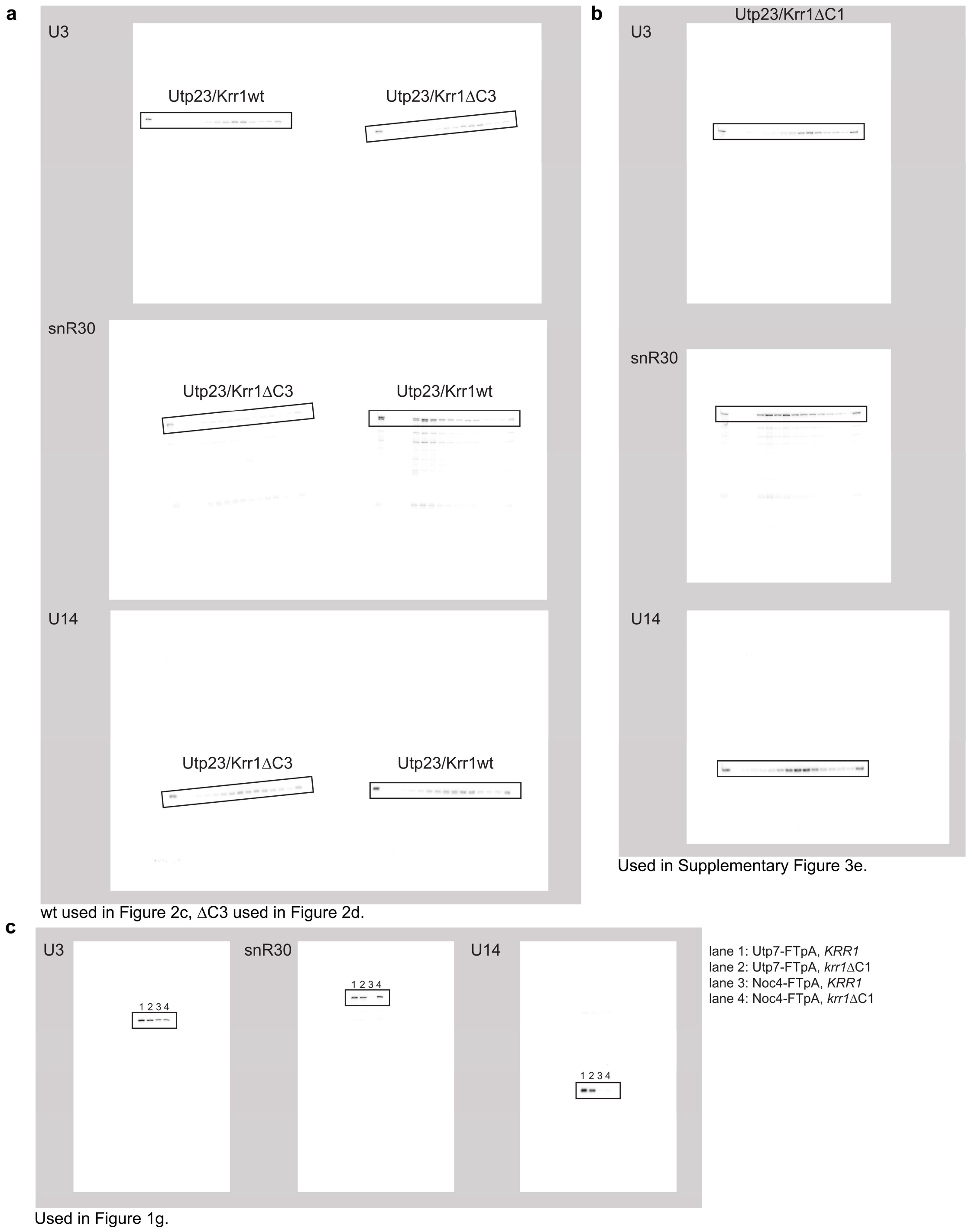

Source Data Figure 3 Uncropped Northern Blots from Main and Supplementary Figures.

Uncropped images of Northern Blots. The line indicates the region cropped for display in the respective figures. a: Figure 2, b: Supplementary Figure 3, c: Figure 1.

a SDC 5-FOA

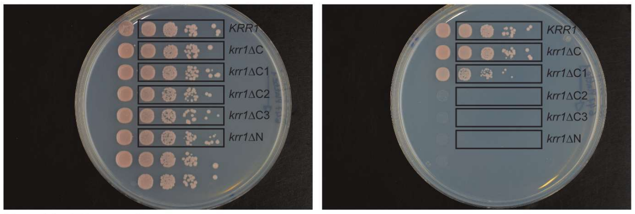

Used in Figure 1c.

b SDC 5-FOA

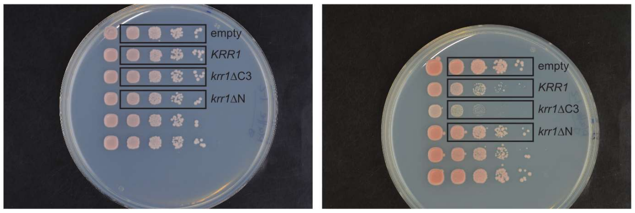

Used in Figure 1d.

c 23°C 30°C 37°C

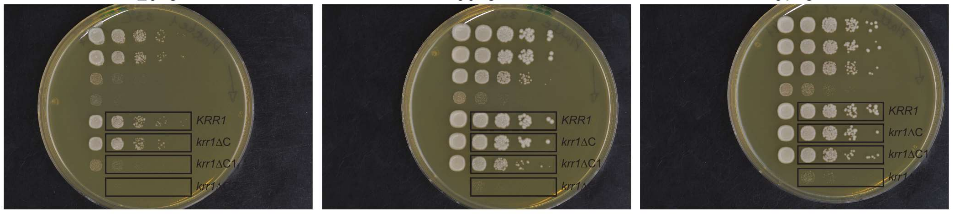

Used in Supplementary Figure 3a.

Source Data Figure 4 Uncropped Dot spots from Main and Supplementary Figures.

Uncropped images of Dotspots. The line indicates the region cropped for display in the respective figures. a, b: Figure 1, c: Supplementary Figure 3.
